# Efficacy of lotilaner (Credelio^™^ chewable tablets) for preventing *Babesia canis* transmission to dogs by infected *Dermacentor reticulatus* ticks

**DOI:** 10.1186/s13071-026-07606-8

**Published:** 2026-08-19

**Authors:** Katrin Deuster, Laura A. Webb, Carin Rautenbach, William E. Anderson, Reinier Zwiegers, Friederike Krämer, Brad Hayes, Lisa Young

**Affiliations:** 1 Elanco Animal Health GmbH, Monheim, Germany; 2 Clinglobal Ltd, Tamarin, Mauritius; 3 Elanco Animal Health , Indianapolis, USA; 4 Q-Vative (Pty) Ltd, Bloemfontein, South Africa; 5 TransMIT GmbH, Giessen, Germany; 6 Elanco Animal Health UK Ltd, Hook, UK

**Keywords:** *Babesia canis*, Canine babesiosis, Credelio™, *Dermacentor reticulatus*, Dog, Ectoparasiticide, Efficacy, Ixodid ticks, Lotilaner, Oral, Ornate dog tick, Prevention, Vector-borne

## Abstract

**Background:**

Canine babesiosis is a clinically significant tick-borne disease, caused by different *Babesia* species. *Babesia canis,* one of the most important and widespread species in Europe, is transmitted by *Dermacentor reticulatus* ticks and can cause potentially fatal canine disease. Prevention of pathogen transmission can be achieved by regular tick control and prophylaxis. The present study evaluated the efficacy of a single oral application of lotilaner (Credelio^™^, Elanco) in dogs for blocking pathogen transmission compared to a negative control group.

**Methods:**

Twenty-four *B. canis*-negative dogs were allocated to three groups (*n *= 8 each). The two treatment groups were treated once orally with Credelio either on Day 0 or 23, while the negative control was sham-dosed accordingly. All dogs were challenged with *B. canis*-infected *D. reticulatus* on Day 28, i.e., 28- or 5-days post-treatment (p.t.), for the lotilaner-treated groups. Tick efficacy was assessed by in situ tick counts 24 and 48 h after infestation (Days 29 and 30) and final tick counts and removals 120 h after infestation (Day 33).

Clinical examinations, hematology (blood smear), serology (IFAT) and blood PCR were performed in all dogs systematically and in case of suspected clinical babesiosis.

**Results:**

A single oral treatment with Credelio 28 or 5 days prior to infestation with *B. canis*-infected *D. reticulatus* resulted in 100% blocking efficacy against *B. canis* transmission, as compared to untreated dogs (*p* < 0.001). The lotilaner-treated dogs were healthy and did not show any clinical or hematological signs of *B. canis* infection. No treated dog seroconverted or showed a positive PCR. The untreated negative controls tested positive for *B. canis* from Day 35 onward. All negative controls developed *B. canis* infection by Day 36 (clinical signs, blood smear, PCR). From Day 43 onward, positive IFAT was also observed.

Lotilaner administered on Day 0 or Day 23 demonstrated tick efficacy of 99.7 and 100%, respectively (Day 33).

**Conclusions:**

This study confirmed a single oral application of lotilaner chewable tablets was highly effective (100%) against *D. reticulatus*, blocking the transmission of *B. canis* to dogs at the beginning as well as at the end of the monthly treatment interval of Credelio.

**Graphical Abstract:**

**Figure:**
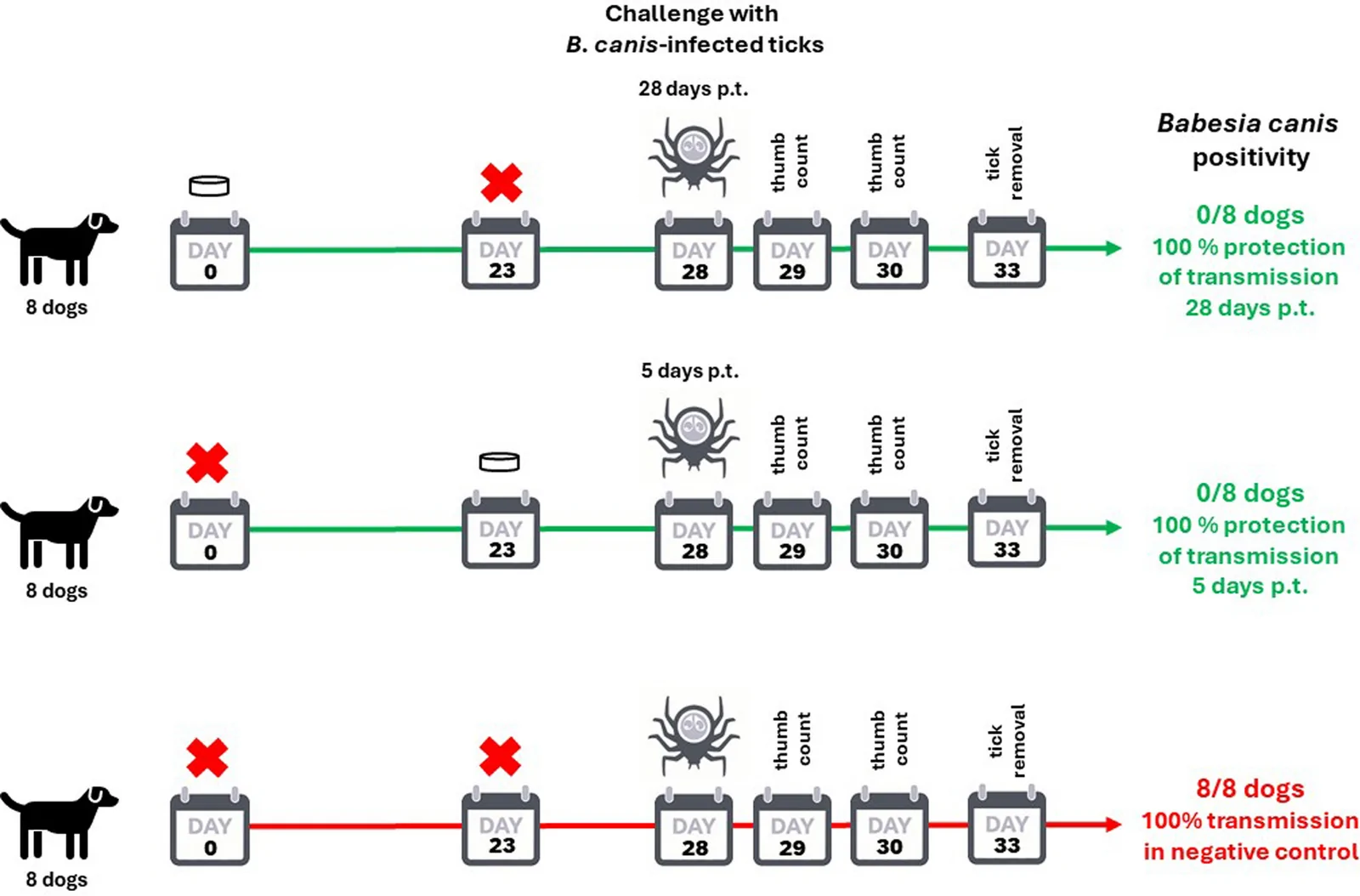

## Background

Pathogens of the genus *Babesia* are primarily transmitted through tick bites, allowing for the infection of a wide range of domestic and wild animals as well as humans [1]. For dogs, several *Babesia* species are recognized, which were formerly differentiated into large and small species by the morphology and size of the protozoan within the red blood cell. Meanwhile, molecular techniques allow the identification of several canine *Babesia* species. The large canine species include *Babesia canis*, *Babesia vogeli* and *Babesia rossi* as distinct species [2] and an additional currently unnamed large-form species described in North Carolina in the United States [3]. Among the small canine *Babesia* species, only three are considered clinically significant: *Babesia gibsoni*, *Babesia conradae* [4, 5], and *Babesia vulpes* n.sp. (formerly named *Babesia* "Spanish dog isolate", *Babesia* "*microti*-like", "*Babesia* (*Theileria*) *annae*", *Babesia* cf. *microti* and *Babesia vulpes*) [6].

Of all these species, *B. canis*, *B. vogeli*, *B. gibsoni* and *B. vulpes* n.sp. are of importance in Europe, mainly due to a widespread distribution of their vectors (*B. canis* and *B. vogeli* with *Dermacentor reticulatus* and *Rhipicephalus sanguineus*, respectively). The large dependence on the geographical distribution of the competent tick vector implies a highly variable geographical spread of *Babesia* spp. infections in Europe (see [7] for more details).

Climatic and ecological changes, national regulations on the management of stray dogs and cats together with an increase in pet travel and translocation of pet animals can influence the epidemiological situation of vector-borne diseases in Europe [8]. Increased importation of infected animals or the spread and establishment of causative agents and their vectors in previously non-endemic areas may cause an increase in frequency of rare diseases in certain areas [8]. Such an expansion of endemic areas has been recorded for various parasitic diseases, among them babesiosis. The latter has been observed more frequently across central Europe in the past few years, emerging from previous endemic regions [8].

*Babesia canis* is the most widely distributed European canine species and is considered to be an ‘infection of central Europe’, principally because of the abundance of *D. reticulatus* in that region [9], spanning from Portugal in the West to Ukraine and Belarus in the East (for detailed maps on *D. reticulatus* see [10]). Details on the occurrence (map and tables) of the other important canine *Babesia* species in Europe can be found in Solano-Gallego et al. [7]. *Dermacentor reticulatus* as vector tick prefers cool and wet climates [11]. The tick occurs in mild, damp open areas that are sparsely covered by trees and bushes, particularly, natural deciduous forests placed near water bodies or large stagnant waters [9]. Ideal habitats are alluvial forests and swamps, where flooding for certain periods can be survived by the tick [12]. Nevertheless, the tick also readily colonizes drier habitats such as fallow and heathland as well as grassland interspersed with bushes or trees and may also occur in urban and suburban areas, such as city parks and fallow land [13–15]. Adult *D. reticulatus* ticks parasitise dogs, while immature individuals feed on wild rodents and are endophilous. Adult *D. reticulatus* are active from late August/September through April/May, only interrupted by low temperatures or snow cover in the winter [10].

During the blood meal of the tick, *Babesia* sporozoites are transmitted from the tick’s salivary gland to the vertebrate host, whereupon the protozoan life cycle is completed within the red blood cells. For *D. reticulatus*, the first developmental stages of *B. canis* in the salivary glands were recorded from the second day onward after tick attachment [16]. Transmission of *B. canis* via infected *D. reticulatus* ticks was recorded at a minimum of 48 h attachment (48–72 h). At 24 h attachment, transmission of *B. canis* has not been successful [17]. However, under experimental conditions, 'pre-activated' *D. reticulatus* ticks (detached 72–88 h post-infestation from one host and re-attached to a second host) transmitted *B. canis* in as little as 8 h [18]. These experimental conditions illustrate transmission after interrupted-feeding from a previous host in contrast to the conditions in Heile et al. [17], testing direct transmission without detachment and interruption.

The process of transmission and completion of the protozoan life cycle within the host’s red blood cells is accompanied by a variety of clinical signs in the canine host, ranging from mild transient illness to acute disease due to severe hemolysis which may rapidly result in death. The clinical outcome depends among others on the *Babesia* species. Furthermore, the dog’s age, immune status and the presence of other infectious diseases may also play a role for clinical signs and disease outcome [19]. Common clinical signs of *Babesia* spp. infection are fever, lethargy, anorexia, pale mucous membranes, weakness, bounding pulse, jaundice, pigmenturia, mild to severe thrombocytopenia, mild to severe regenerative anemia due to hemolysis, bilirubinemia, bilirubinuria, and hemoglobinuria [7]. In case of *B. canis* infection, the disease outcome is moderate to severe, and the course of infection is acute (see [7] for more details).

Canine babesiosis is a common and clinically significant tick-borne hemoprotozoan disease with treatment depending on the *Babesia* species. Dogs diagnosed with an infection with *B. canis* or *B. vogeli* will generally manifest a complete recovery following treatment [20, 21]; however, there have also been reports of a high mortality rate (53%, 8 out of 15) in dogs with *B. canis*-induced babesiosis during the first 24–48 h after clinical presentation and treatment [22].

The clinical manifestations and potential severe prognosis both emphasize the importance of preventing pathogen transmission. In this context, tick control is paramount. In accordance with ESCCAP guidelines, tick prophylaxis should cover the entire period of tick activity. Depending on risk level and local legislation, this may involve regular tick checks and/or acaricidal treatment [8]. With a documented transmission time of at least 48 h after tick attachment [17] and 8 h in detached and re-attached ticks [18], acaricidal compounds should be fast-acting to block *Babesia* transmission.

The biological basis for interrupting *B. canis* transmission was established in foundational studies characterizing the maturation of the parasite within the vector. Early electron microscopy and life-cycle studies demonstrated that *Babesia* sporozoites exist in a dormant state within the tick salivary glands and require the physiological stimulus of a blood meal to undergo final transformation into infective stages [16, 23]. This 'activation' period provided the theoretical window for transmission-blocking. Experimental validation of this concept progressed in the 2000s, as researchers shifted focus from simple acaricidal efficacy (tick mortality) to the prevention of pathogen inoculation. Experimental models using topical collars and spot-on formulations were among the first to demonstrate that tick knockdown could successfully block *Babesia* transmission under controlled infestation challenges [24–28]. Building upon these principles, the development of the isoxazoline class has further refined transmission-blocking capabilities through systemic activity and rapid speed of kill. Recent investigations have demonstrated that this class can consistently block the transmission of *B. canis* by eliminating *D. reticulatus* well within the established pre-patent period [29–35].

In the current study, treatment was performed once orally with lotilaner chewable tablets (Credelio^™^; Elanco, Indianapolis, IN, USA). Lotilaner, a most recent addition to the class of ectoparasiticidal isoxazolines, is registered for monthly administration to cats and dogs. The compound provides quick onset and consistent efficacy against flea and tick infestations in dogs for at least 1 month [36–38], based on its 2 h T_MAX_ and 30.7-day terminal half-life under fed conditions [39].

Studies in both cats and dogs have demonstrated lotilaner’s safety and its fast, sustained efficacy against a range of feline and canine ectoparasites, including several tick species (*Amblyomma americanum*, *Amblyomma cajennense*, *Dermacentor reticulatus*, *Dermacentor variabilis*, *Haemaphysalis longicornis*, *Ixodes hexagonus*, *Ixodes holocyclus*, *Ixodes ricinus*, *Ixodes scapularis* and *Rhipicephalus sanguineus*) [40–48], fleas (*Ctenocephalides felis* and *Ctenocephalides canis*) [36, 37, 49–54] and mites (*Demodex* spp.) [55].

The objective of this study was to determine the efficacy of a single dose of lotilaner (Credelio chewable tablets for dogs) in blocking transmission of *B. canis* from infected *D. reticulatus* ticks to dogs by killing the ticks before transmission of *B. canis* occurred. For this, different tick infestation and treatment scenarios were performed with efficacy assessments either at the beginning (5-day post-treatment [p.t.]) or at the end (28-day p.t.) of the regular treatment interval of Credelio. Dogs were screened for *B. canis* infection using hematological, serological and molecular test systems and were examined regularly with a special focus on signs of clinical babesiosis.

## Methods

### Study design

The study was conducted in accordance with standards of Good Clinical Practice (VICH Guideline 9) [56] as well as EMA and W.A.A.V.P. guidelines on parasiticide efficacy related to both tick infestation and vector-borne pathogen transmission in dogs and cats [57–60]. The study was a masked, randomized, complete block design, negatively controlled efficacy study to evaluate the effectiveness of lotilaner in form of Credelio chewable tablets for dogs challenged with *B. canis*-infected *D. reticulatus* ticks under laboratory conditions, compared to a sham-dosed control group. Blinding of the study personnel was accomplished by separation of study roles. Dogs in Group 1 (*n *= 8 dogs) were sham-dosed on Days 0 and 23 (negative control); dogs in Group 2 (*n* = 8 dogs) were treated with Credelio on Day 0 and sham-dosed on Day 23 (Credelio Day 0) to evaluate efficacy of the treatment at the end of the protection period of lotilaner; dogs in Group 3 (*n* = 8 dogs) were sham-dosed on Day 0 and treated with Credelio on Day 23 (Credelio Day 23) to evaluate efficacy of the treatment at the beginning of the protection period of lotilaner. All dogs were infested with *B. canis*-infected *D. reticulatus* ticks on Day 28. *Babesia* infection was assessed using hematology (blood smear), serological and molecular tests and clinical examination.

### Animal details

Dogs included into the study were purpose-bred Beagles and cross-breeds of both sexes (17 Beagles, 7 cross-breeds; 11 males, 13 females), between 18 and 90 months as of Day 0 and weighing between 11.8 and 26.4 kg (Day -4). After physical examination, where all dogs were confirmed to be healthy, a 7-day acclimatization period started. Within this period, dogs were challenged by experimental infestation with uninfected *D. reticulatus* ticks on Day -6 to evaluate their suitability for tick infestation and for ranking and allocation purposes. Blood samples to confirm *B. canis* naivety were taken twice prior to Day 0 and tested via immunofluorescent antibody test (IFAT).

For the duration of the study, dogs were housed individually in appropriately sized units with visual and auditory but no physical contact between neighboring animals. Animal enrichment (toys or chewing devices) was provided to each dog and replenished weekly. All dogs were fed with standard, commercially available dry food for dogs, according to the manufacturer’s recommendation, and had free access to potable municipal water.

None of the dogs included into the study had been treated with drugs which could have interfered with the objectives of the study prior to study start. None of the dogs were pregnant, lactating or intended for breeding purposes.

For group randomization, all study animals were ranked based on individual pre-treatment (Day -4) live attached adult *D. reticulatus* counts. Subsequently, ranked animals were blocked into eight blocks of three dogs each. Ascending order of animal ID was used to break ties. Within blocks, dogs were randomly allocated to the three study groups (negative control, Credelio Day 0 and Credelio Day 23).

### Animal health

All dogs were deemed clinically healthy upon physical examination on Day -7. General health observations were performed at least once daily throughout the study duration. Clinical examinations for signs of babesiosis were conducted on Day 27 (prior to tick infestation on Day 28) and then weekly until the end of the study (Days 35, 43, 49, 56). Body temperatures were measured daily starting on Day 33 until the end of the study (except for days of weekly clinical examination).

### Credelio treatment

Dogs were treated in the fed state. Dogs received Credelio tablets to achieve as close to the minimum target dosage of 20 mg/kg of lotilaner without underdosing, resulting in dosages of lotilaner between 20.234 and 24.457 mg/kg body weight. Doses were determined based on the individual body weights recorded prior to treatment (Days -4 and 22). Before and after treatment health observations were performed in all dogs hourly for 3 h (± 15 min). Handling and sham-dosing procedures for dogs in the negative control group and for those receiving sham doses in the two treatment groups were identical to those used for dogs receiving Credelio treatment. The following treatment groups existed: Credelio Day 0 group: oral lotilaner administration on Day 0 and sham dose on Day 23; Credelio Day 23 group: sham dose on Day 0 and oral lotilaner administration on Day 23; negative control group: sham-dosed on Day 0 and ay 23.

### Anti-babesial rescue treatment

For dogs with abnormally high body temperatures (> 39.4 °C) and clinical signs commonly associated with babesiosis, two blood smears were prepared and evaluated for *B. canis*. In case of a positive *B. canis* result on a blood smear, blood was taken for PCR analysis, and the dog underwent rescue treatment. This included the following concomitant treatments according to manufacturer recommendations: Berenil^®^ R.T.U. s.c. injection (diminazene; MSD, India), Forray^®^ 65 s.c. injection (imidocarb dipropionate; MSD [Pty] Ltd, South Africa), Kyro B + Liver (vitamin B complex s.c. injection with liver extract and choline chloride; Kyron Laboratories [Pty] Ltd, South Africa), Be-Tabs Prednisone 5 mg tablets (prednisone; Ranbaxy Pharmaceuticals [Pty] Ltd, South Africa), Cerenia s.c. injection (maropitant; Zoetis South Africa [Pty] Ltd, South Africa), Heptonic (nutritional supplement, Kyron Laboratories [Pty] Ltd, South Africa), and Hill’s a/d (dietetic pet food, Hill’s Pet Nutrition Manufacturing s.r.o., Italy).

Dogs receiving rescue treatment remained in the study for the purpose of monitoring subsequent blood samples with IFAT and PCR.

### Tick infestations and assessments

A laboratory-maintained *D. reticulatus* tick isolate (European origin) was used for tick infestations on Day -6 and Day 28. Ticks used on Day -6 for tick retention assessment and group randomization were uninfected. Ticks used for challenging tick protection and prevention of pathogen transmission on Day 28 were infected with *B. canis*. On Day 28, each dog was infested with approximately 50 unfed *D. reticulatus* ticks (*B. canis* infectivity of 23%; 1:1 male to female ratio).

For tick infestation, dogs were sedated with Domitor^®^ (medetomidine; Zoetis South Africa [Pty] Ltd, South Africa). Ticks were released onto the back of the dogs and allowed to disperse. Dogs were placed into infestation chambers measuring 50×50x90 cm for a period of approximately 2 h to facilitate tick infestation. After the tick infestation procedure, sedation was reversed with Antisedan^®^ (atipamezole hydrochloride; Zoetis South Africa [Pty] Ltd, South Africa) as required.

On Day -4, i.e., 2 days after infestation, ticks were counted and removed from all dogs. Following the tick challenge on Day 28, tick in situ thumb counts without removal were performed on each dog on Days 29 and 30 (24 and 48 h post-infestation [± 1 h]). To allow sufficient time for potential *B. canis* transmission, all remaining ticks on each dog were counted and removed on Day 33 (i.e., 5 days after tick infestation, as close as possible to 120 ± 2 h after infestation). Study personnel conducting tick infestation, tick in situ counts and removal were blinded to the treatment status of each dog.

### Serological, molecular and hematological analysis for detection of *B. canis* infection

Blood samples for serum analysis of *B. canis* antibodies were collected at study start on Day -7, on Day 27 and then weekly until the end of the study (Days 35, 43, 49 and 56). Screening for *B. canis* antibodies by IFAT was performed using MegaFLUO^®^ BABESIA canis IFA test kit (MEGACOR Diagnostik GmbH, Germany).

Blood samples for PCR analysis to detect *B. canis* DNA were collected on the same days as described above for serology with the exception of the Day -7 timepoint. Total genomic DNA was isolated from whole blood samples, using a commercial genomic DNA isolation kit (GeneJET Genomic DNA Purification Kit, Thermo Fisher Scientific Inc., Waltham, MA, USA). DNA isolation and PCR analysis were performed according to procedures described in Troskie et al. [61], using *B. canis* specific primers. A PCR product of approximately 128 bp indicated the presence of *B. canis* DNA in the sample.

Blood samples for hematological examination, consisting of two duplicate blood smears, were taken whenever daily health observations, clinical examinations or body temperature suggested clinical babesiosis in a study dog. For this, a small amount of capillary blood taken from the periphery of the ear was used. A clean microscope slide was used to collect that drop of blood. Subsequently, the blood drop was smeared across the slide using the wedge technique or "push" technique to create a thin, feathered blood layer. After fixation using Diff-Quik stain, blood smears were microscopically examined.

In case of a positive blood smear result, PCR analysis as described above was subsequently performed to confirm infection with *B. canis*.

### Efficacy evaluation and data analysis

The software package SAS^®^ v9.4 (SAS Institute Inc., Cary, NC, USA) [62] was used for all statistical analyses with the individual dog as experimental unit. All hypotheses were tested at a two-sided 0.05 level of significance. In the descriptions below, “treated group” refers to treatment Group 1 (receiving Credelio on Day 0) or treatment Group 2 (receiving Credelio on Day 23).

For the primary endpoint, blocking efficacy of *B. canis* transmission, treatment was considered effective if the following criteria were met: 

The negative control group had adequate *B. canis* infection, defined as at least six dogs that were ever positive.

Each treated group had percentage blocking efficacy ≥ 90%, where:

$$Blocking\,efficacy\,\left( \% \right)\, = \,100\,\, \times \,\left( {T_{c} \, - \,T_{T} } \right)\,/T_{C}$$

and T_C_ and T_T_ are the proportions of dogs in the negative control group and treated group, respectively, that were infected with *B. canis* by the end of the study.

The proportion of infected dogs in each treated group was significantly lower than that in the negative control group, assessed using Fisher’s exact test.

A dog was considered infected with *B. canis* if it tested positive on at least one IFAT and at least one PCR test by end of the study. Any dog rescue treated prior to IFAT or PCR assessment was considered positive for infection with *B. canis*.

Efficacy against *D. reticulatus* ticks was evaluated using live attached tick counts. Treatment was considered effective for the control of *D. reticulatus* when the following criteria were met:

- The negative control group had adequate infestation, defined as ≥ 25% retention of total live ticks in at least six dogs, for the Day 33 tick counts.
- Each treated group had a percentage efficacy ≥ 90% using least squared (LS) means calculated from a linear mixed model of live attached tick counts collected on Day 33, as described below.
- Each treated group had a significantly lower number of live attached ticks than that in the negative control group, assessed using a linear mixed model of tick counts collected on Day 33, as described below.

Separate linear mixed models, one for each treated group, were analyzed, with each live attached tick counts as the response variable, treatment (i.e., treated v. negative control) as a fixed effect, and room and block as random effects. The treatment effect was used to determine statistical significance.

The percentage efficacy of each treated group was calculated by comparing the LS means between the treated dogs and the control dogs from the respective linear mixed model, as follows:

$$Efficacy\,\,\left( \% \right)\, = \,100\,\, \times \,\left( {LS\,Mean_{Control} \, - \,LS\,Mean_{Treated} } \right)\,/\,LS\,Mean_{Control}$$

As the models used untransformed counts, the LS means are equivalent to arithmetic means (AM). In similar fashion, percentage efficacy using LS means was also calculated for in situ counts collected on Days 29 and 30, but statistical significance was not assessed for these counts. In addition, percentage efficacy using geometric means (GM) was calculated for Days 29, 30 and 33.

## Results

No treatment-related serious adverse events were observed in any of the dogs during the study. Non-serious gastrointestinal adverse events (diarrhea) were observed in two dogs (one Credelio Day 0 dog, one Credelio Day 23 dog) at 2 h ± 15 min p.t. on Day 0 and 23, respectively. A relation to treatment was considered likely. Both animals recovered at the 3 h ± 15 min post-observation timepoint. All negative control dogs exhibited abnormal clinical signs associated with babesiosis (e.g., high body temperature, bloody/dark urine, pale mucous membranes, anorexia, lethargy) starting on Days 35 and 36, respectively. All clinical signs were completely resolved by Day 43.

### Transmission-blocking efficacy

The primary endpoint of the study was the blocking efficacy of *B. canis* transmission based on the infection status of each treatment group at the end of the study (Day 56). All eight negative control dogs tested positive for *B. canis* by at least one blood smear, IFAT and PCR. In contrast, all eight dogs in both the Credelio Day 0 and Credelio Day 23 groups remained negative on every test, with no incidence of infection despite exposure to *Babesia*-infected *D. reticulatus* ticks between Days 28 and 33.

This resulted in 100% blocking efficacy for the Credelio Day 0 and Credelio Day 23 groups. The incidence of *B. canis* in each of the lotilaner-treated groups was significantly lower than in the negative control group (*p* < 0.001). For details, see Tables 1 and 2 as well as the paragraphs below.

**Table 1:** Results of diagnostic tests for *Babesia canis* infection in dogs and tick count data after infestation with *Dermacentor reticulatus* ticks on Day -6 and Day 28

	Animal ID	*Babesia* diagnosis (Day recorded)			Total live attached tick counts			
		Blood smears in duplicate (x/x)^a^	PCR^b^	IFAT^c^	Day -4	Day 29	Day 30	Day 33
Credelio Day 0	1	n.a	–	–	27	15	0	0
	2	n.a	–	–	32	1	0	0
	3	n.a	–	–	18	16	9	1
	4	n.a	–	–	26	1	0	0
	5	n.a	–	–	21	18	11	0
	6	n.a	–	–	19	19	11	0
	7	n.a	–	–	14	14	1	0
	8	n.a	–	–	23	4	0	0
	Mean^d^				22.5	11.0	4.0	0.1
Credelio Day 23	9	n.a	–	–	21	6	2	0
	10	n.a	–	–	13	2	0	0
	11	n.a	–	–	19	4	0	0
	12	n.a	–	–	27	2	0	0
	13	n.a	–	–	24	3	0	0
	14	n.a	–	–	28	5	0	0
	15	n.a	–	–	42	0	0	0
	16	n.a	–	–	18	4	0	0
	Mean^d^				24.0	3.3	0.3	0
Negative control	17	+ /– (35)	+ (35)	+ (43, 49, 56)	21	31	31	37
	18	+ / + (36)	+ (35, 36)	+ (43, 49, 56)	13	29	30	32
	19	+ / + (35)	+ (35)	+ (43, 49, 56)	28	23	27	40
	20	+ / + (35)	+ (35)	+ (43, 49, 56)	16	20	24	34
	21	+ / + (35)	+ (35)	+ (43, 49, 56)	27	17	23	46
	22	+ / + (36)	+ (35, 36)	+ (43, 49, 56)	30	19	20	44
	23	+ / + (35)	+ (35)	+ (43, 49, 56)	21	34	33	39
	24	+ / + (35)	+ (35)	+ (43, 49, 56)	22	28	34	41
	Mean^d^				22.3	25.1	27.8	39.1

n.a. = not applicable; +  = positive test result; −  = negative test result

^a^Performed in case of suspected *Babesia* infection during general health observations and clinical examinations; once positive, PCR was performed for confirmation and subsequent rescue treatment initiated

^b^Minimum days performed in all dogs: Days 27, 35, 43, 49, 56; Day 36 additionally for two control dogs

^c^Minimum days performed in all dogs: Days -7, 27, 35, 43, 49, 56

^d^Mean = arithmetic mean

**Table 2:** Total number of dogs with positive incidence of *Babesia canis* infection by the different diagnostic tests and corresponding blocking efficacy (%) of Credelio administered once orally to dogs (Day 0 or Day 23) against experimentally induced transmission of *B. canis* by infected *Dermacentor reticulatus* ticks (infestation on Day 28)

Group	Positive incidence *n*/*N* (%)				Blocking efficacy (%)^a^
	Blood smears	PCR	IFAT	Overall^b^	
Credelio Day 0	n.a	0/8 (0)	0/8 (0)	0/8 (0)	100 *p* < 0.001*
Credelio Day 23	n.a	0/8 (0)	0/8 (0)	0/8 (0)	100 *p* < 0.001*
Negative control	8/8 (100)	8/8 (100)	8/8 (100)	8/8 (100)	

*n* = number of dogs with positive incidence; *N* = number of dogs within a group

n.a. = not applicable

^b^Positive incidence recorded if at least one positive IFAT and one positive PCR test had been recorded after Day 28

^*^Fisher’s exact test comparing overall positive incidence of treated group to that of negative control group, statistically significant

### Serological results

All dogs were IFAT negative at study start (day -7) as well as on Days 27 and 35. From Day 43 until study end (Days 43, 49, 56), all eight negative control dogs consistently showed positive IFAT results, indicating seroconversion. Conversely, none of the Credelio-treated dogs (Credelio Day 0 and Day 23 groups) had a positive IFAT at any point during the study, remaining seronegative (see Tables 1 and 2).

### Molecular results

All dogs were PCR negative for *B. canis* on Day 27, prior to the Day 28 tick infestation. By Day 35, all negative control dogs tested PCR positive, with blood smear positivity on Days 35 and 36, respectively, confirmed. Following rescue treatment initiated on Day 35 or 36, these eight negative control dogs tested PCR negative again until study end (Days 43, 49, 56). In contrast, none of the Credelio-treated dogs (Credelio Day 0 and Day 23 groups) ever yielded a positive PCR result throughout the study (see Tables 1 and 2).

### Clinical examinations and blood smears

Six negative control dogs were observed with abnormal clinical signs associated with babesiosis during the weekly assessment on Day 35. The remaining two dogs followed 1 day later with similar clinical signs. In total, six negative control dogs presented with high body temperature (≥ 39.9 °C) on Day 35 and Day 36, respectively. Temperatures normalized the following day. Three negative control dogs were observed to have bloody/dark urine between Days 35 and 37. Pale mucous membranes occurred in four dogs, anorexia in three dogs and lethargy in one dog. All of these clinical signs completely resolved after rescue treatment by Day 43.

In dogs exhibiting clinical signs indicative of babesiosis, at least one of the two blood smears performed on Day 35 and Day 36 was positive (see Table 1).

### Tick efficacy

Efficacy of Credelio against *D. reticulatus* ticks was evaluated using live attached tick counts. Efficacy was assessed at the end of the treatment period for the Credelio Day 0 group (29-, 30- and 33-day p.t.) and at the beginning of the treatment period for the Credelio Day 23 group (6-, 7- and 10-day p.t.). Individual live attached tick counts and LS means are listed in Table 1, treatment group comparisons are provided in Table 3. In summary, a single treatment with Credelio significantly (*p* < 0.001) reduced *D. reticulatus* infestations in dogs at 33- or 10-day p.t., resulting in 99.7% (Credelio Day 0) and 100% efficacy (Credelio Day 23), respectively (Table 3).

**Table 3:** Summary of total live attached tick counts and corresponding efficacy (%) of Credelio administered once orally to dogs (Day 0 or Day 23) against experimentally induced infestations with *Dermacentor reticulatus* on Day 28

Day	Treatment group	Live attached tick count LS mean (GM)	% Efficacy based on LS means (GM)	*P* value**
29	Negative control	25.1 (24.5)	n.a	n.a
	Credelio Day 0	11.0 (7.5)	56.2 (68.8)	*
	Credelio Day 23	3.3 (2.7)	87.1 (88.8)	*
30	Negative control	247.8 (27.3)	n.a	n.a
	Credelio Day 0	7.0 (1.7)	85.6 (93.8)	*
	Credelio Day 23	0.3 (0.1)	99.1 (99.5)	*
33	Negative control	39.1 (38.9)	n.a	n.a
	Credelio Day 0	0.1 (0.1)	99.7 (99.8)	< 0.001
	Credelio Day 23	0 (0)	100 (100)	< 0.001

n.a. = not applicable

^*^ *P* value not calculated on Days 29 and 30

^**^ Calculated for LS means only

## Discussion

The present study demonstrates that Credelio (lotilaner) chewable tablets successfully blocked the transmission of *B. canis* to dogs by effectively killing infected *D. reticulatus* ticks. Dogs treated with a minimum dosage of 20 mg/kg were protected against transmission when challenged either 5- or 28-days post-administration. No treatment-related serious adverse events occurred, supporting the safety of the product in the evaluated population. Two dogs experienced transient diarrhea approximately 2-h post-administration; however, this did not seemingly impact the absorption or pharmacokinetics of the drug, as both animals were successfully protected against *B. canis*. These findings align with our broader clinical data, where self-limiting gastrointestinal signs, such as vomiting or diarrhea, does not compromise product performance.

The validity of the infection model was confirmed by the 100% infection rate in the negative control group. All eight sham-dosed dogs developed clinical babesiosis, supported by positive blood smears, IFAT, and PCR. Given that *B. canis* is a prominent and potentially fatal pathogen in Europe—often with high mortality rates within the first 24–48 h of clinical presentation [22]—the ability to prevent transmission entirely is a critical clinical objective.

The prevention of pathogen transmission relies on interrupting the biological "window of opportunity" during tick feeding [63]. Pathogens like *B. canis* often require a period of "reactivation" and translocation within the tick after feeding begins. While the exact timing of *B. canis* transmission remains a subject of debate, studies have shown that while 48–72 h of attachment is typically required [17], "pre-activated" ticks (those with prior interrupted feeding) can transmit the pathogen in as little as 8 h [18]. This underscores the necessity for an ectoparasiticide with a rapid speed of kill and sustained residual efficacy [63, 64].

Lotilaner, a member of the isoxazoline class, is characterized by its rapid onset of action. Previous studies have shown lotilaner begins killing *I. ricinus* within 4 h in dogs and achieves near-total efficacy against *D. reticulatus* within 48 h over a 35-day period [38, 44]. Our findings confirm that this fast-acting property translates into 100% blocking efficacy against *B. canis* reducing the risk of transmission throughout the entire treatment interval.

To address the risk posed by pre-activated ticks—which can transmit pathogens significantly faster than the standard 48-h window—the mechanism of protection likely extends beyond tick mortality. Lotilaner acts as a potent antagonist of gamma-aminobutyric acid (GABA)-gated chloride channels, inducing rapid neurological hyperexcitation and paralysis [65]. Research into these "pre-lethal effects" indicates that lotilaner causes discoordination and the cessation of effective feeding behavior well before the tick dies [65]. In the context of *B. canis*, which requires coordinated muscular contractions for blood ingestion and salivary secretion, this immediate neurological disruption likely inhibits the translocation of the pathogen. By neutralizing the tick’s feeding apparatus shortly after the first blood meal, lotilaner effectively closes the transmission window.

It is important to acknowledge that laboratory-controlled models have inherent limitations compared to real-world field challenges, such as the varied physical activities of dogs and the interrupted-feeding patterns of wild ticks. However, the documented pre-lethal effects of lotilaner [65] suggest that the rapid cessation of feeding occurs even before tick mortality. This mechanism provides a robust biological bridge between standardized laboratory observations and the erratic feeding behaviors often encountered in the field.

While the transmission-blocking efficacy of lotilaner has been previously reported [29], the present study provides a distinct and incremental contribution through a refined experimental design. By employing a staggered treatment schedule (Day 0 and Day 23) followed by a simultaneous challenge on Day 28, we were able to conduct a head-to-head comparison of efficacy at both the beginning and end of the treatment interval using the exact same batch of infected ticks. This eliminated the confounding variable of tick batch variability, providing a more rigorous validation of the product's sustained speed of kill.

Our results align with findings for other isoxazolines, such as afoxolaner, fluralaner, and sarolaner, which have also demonstrated high blocking efficacy against various pathogens [29– 35]. Collectively, these data reinforce the conclusion that Credelio fulfills the requirement for a very fast onset of action as the principal feature of an ectoparasitic drug intended to block vector-borne disease transmission.

## Conclusions

This study confirmed a single oral application of lotilaner chewable tablets (Credelio), administered at a minimum dosage of 20 mg/kg, was safe and highly effective against *D. reticulatus,* blocking the transmission of *B. canis* to dogs. Blocking efficacy (100%) was demonstrated at both the beginning (5-day p.t.) and end (28-day p.t.) of the monthly treatment interval. Credelio-treated dogs remained negative until the end of the study (Day 56), while all negative control dogs were still IFAT positive on Day 56. Credelio chewable tablets may thus be used for the reduction in the risk of canine babesiosis and the protection of dogs in endemic regions.

## Data Availability

The dataset summarizing and supporting the conclusions of this article are included within the article.

## Abbreviations

bp: Base pair
CVMP: Committee for Veterinary Medicinal Products
EMA: European Medicines Agency
EMEA: European Medicines Evaluation Agency
ESCCAP: European Scientific Counsel Companion Animal Parasite
GCP: Good Clinical Practices
IFAT: Immunofluorescent antibody test
LS means: Least squared means
PCR: Polymerase chain reaction
p.t.: Post-treatment
VICH: Veterinary International Conference on Harmonization
W.A.A.V.P: World Association for the Advancement of Veterinary Parasitology

## References

[1] Schnittger L, Rodriguez AE, Florin-Christensen M, Morrison DA. *Babesia*: a world emerging. Infect Genet Evol. 2012;12:1788–809. 22871652 10.1016/j.meegid.2012.07.004

[2] Carret C, Delbecq S, Labesse G, Carcy B, Precigout E, Moubri K, et al. Characterization and molecular cloning of an adenosine kinase from *Babesia canis rossi*. Eur J Biochem. 1999;265:1015–21. 10518797 10.1046/j.1432-1327.1999.00806.x

[3] Birkenheuer AJ, Neel J, Ruslander D, Levy MG, Breitschwerdt EB. Detection and molecular characterization of a novel large *Babesia* species in a dog. Vet Parasitol. 2004;124:151–60. 15381295 10.1016/j.vetpar.2004.07.008

[4] Kjemtrup AM, Conrad PA. A review of the small canine piroplasms from California: *Babesia conradae* in the literature. Vet Parasitol. 2006;138:112–7. 16522352 10.1016/j.vetpar.2006.01.045

[5] Kjemtrup AM, Wainwright K, Miller M, Penzhorn BL, Carreno RA. *Babesia conradae*, sp. nov., a small canine *Babesia* identified in California. Vet Parasitol. 2006;138:103–11. 16524663 10.1016/j.vetpar.2006.01.044

[6] Baneth G, Cardoso L, Brilhante-Simões P, Schnittger L 2019 Establishment of *Babesia vulpes* n. sp. (Apicomplexa: Babesiidae), a piroplasmid species pathogenic for domestic dogs. Parasit Vectors 12 129. 10.1186/s13071-019-3385-z PMC6434798 30909951

[7] Solano-Gallego L, Sainz Á, Roura X, Estrada-Peña A, Miró G. A review of canine babesiosis: the European perspective. Parasit Vectors. 2016;11:336. 10.1186/s13071-016-1596-0 PMC4902949 27289223

[8] European Scientific Counsel Companion Animal Parasites (ESCCAP). Control of Vector-Borne Diseases in Dogs and Cats. Guideline 05 Fifth Edition. 2024. https://www.esccap.org/uploads/docs/32ir16g1_0775_ESCCAP_Guideline_GL5_20241203_1p.pdf Accessed 03 Feb 2026.

[9] Karbowiak G. The occurrence of the *Dermacentor reticulatus* tick – its expansion to new areas and possible causes. Ann Parasitol. 2014;60:37–47. 24930245

[10] Rubel F, Brugger K, Pfeffer M, Chitimia-Dobler L, Didyk YM, Leverenz S, et al. Geographical distribution of *Dermacentor marginatus* and *Dermacentor reticulatus* in Europe. Ticks Tick Borne Dis. 2016;7:224–33. 26552893 10.1016/j.ttbdis.2015.10.015

[11] Petney TN, Pfaeffle MP, Skuballa JD. An annotated checklist of the ticks (Acari: Ixodida) of Germany. System Appl Acarol. 2012;17:115–70.

[12] Nosek J. The ecology and public health importance of *Dermacentor marginatus* and *D. reticulatus* ticks in Central Europe. Folia Parasitol. 1972;19:93–102. 4670812

[13] Gilot B, Pautou G, Immler R, Moncada E 1973 Biotopes suburbains á *Dermacentor reticulatus* (Fabricius, 1794) (Ixodea). Etude préliminaire. [Suburban biotype of *Dermacentor reticulatus* (Fabricius, 1794) (Ixodea). Preliminary study.] Rev Suisse Zool. 80 411–30 4748108

[14] Zahler M, Steffen T, Lutz S, Hähnel W, Rinder H, Gothe R 2000 *Babesia canis* and *Dermacentor reticulatus* in Munich: a new endemic focus in Germany (in German). Tierarztl Prax K H. 28 116–20.

[15] Dautel H, Dippel C, Oehme R, Hartelt K, Schettler E. Evidence for an increased geographical distribution of *Dermacentor reticulatus* in Germany and detection of *Rickettsia* sp. RpA4. Int J Med Microbiol. 2006;296:149–56. 16524777 10.1016/j.ijmm.2006.01.013

[16] Schein E, Mehlhorn H, Voigt WP. Electron microscopical studies on the development of *Babesia canis* (Sporozoa) in the salivary glands of the vector tick *Dermacentor reticulatus*. Acta Trop. 1979;36:229–41. 43086

[17] Heile C, Hoffman-Köhler P, Wieman A, Schein E 2007 Transmission time of tick borne disease agents in dogs: *Borrelia, Anaplasma, Ehrlichia* and *Babesia*. Der Praktische Tierarzt. 88 584–90

[18] Varloud M, Liebenberg J, Fourie J. Early *Babesia canis* transmission in dogs within 24 h and 8 h of infestation with infected pre-activated male *Dermacentor reticulatus* ticks. Parasit Vectors. 2018;11:41. 29343275 10.1186/s13071-018-2637-7 PMC5772715

[19] Irwin PJ. Canine babesiosis: from molecular taxonomy to control. Parasit Vectors. 2009;2:S4. 19426443 10.1186/1756-3305-2-S1-S4 PMC2679396

[20] Irwin PJ, Hutchinson GW. Clinical and pathological findings of *Babesia* infection in dogs. Aust Vet J. 1991;68:204–9. 1888313 10.1111/j.1751-0813.1991.tb03194.x

[21] Bourdoiseau G. Canine babesiosis in France. Vet Parasitol. 2006;138:118–25. 16507334 10.1016/j.vetpar.2006.01.046

[22] Eichenberger RM, Riond B, Willi B, Hofmann-Lehmann R, Deplazes P. Prognostic markers in acute *Babesia canis* infections. J Vet Intern Med. 2016;30:174–82. 26727465 10.1111/jvim.13822 PMC4913656

[23] Shortt HE. *Babesia canis*: the life cycle and laboratory maintenance in its arthropod and mammalian hosts. Int J Parasitol. 1973;3:119–48. 4196317 10.1016/0020-7519(73)90019-2

[24] Jongejan F, Fourie JJ, Chester ST, Manavella C, Mallouk Y, Pollmeier MG, et al. The prevention of transmission of *Babesia canis canis* by *Dermacentor reticulatus* ticks to dogs using a novel combination of fipronil, amitraz and (S)-methoprene. Vet Parasitol. 2011;179:343–50. 21777737 10.1016/j.vetpar.2011.03.047

[25] Fourie JJ, Stanneck D, Jongejan F. Prevention of transmission of *Babesia canis* by *Dermacentor reticulatus* ticks to dogs treated with an imidacloprid/flumethrin collar. Vet Parasitol. 2013;192:273–8. 23158840 10.1016/j.vetpar.2012.10.017

[26] Navarro C, Reymond N, Fourie J, Hellmann K, Bonneau S. Prevention of *Babesia canis* in dogs: efficacy of a fixed combination of permethrin and fipronil (Effitix®) using an experimental transmission blocking model with infected *Dermacentor reticulatus* ticks. Parasit Vectors. 2015;8:32. 25595325 10.1186/s13071-015-0645-4 PMC4302435

[27] Jongejan F, De Vos C, Fourie JJ, Beugnet F. A novel combination of fipronil and permethrin (Frontline Tri-Act®/Frontect®) reduces risk of transmission of *Babesia canis* by *Dermacentor reticulatus* and of *Ehrlichia canis* by *Rhipicephalus sanguineus* ticks to dogs. Parasit Vectors. 2015;8:602. 26586365 10.1186/s13071-015-1207-5 PMC4653891

[28] Dumont P, Fourie JJ, Soll M, Beugnet F. Repellency, prevention of attachment and acaricidal efficacy of a new combination of fipronil and permethrin against the main vector of canine babesiosis in Europe, Dermacentor reticulatus ticks. Parasit Vectors. 2015;8:50. 25622802 10.1186/s13071-015-0682-z PMC4316400

[29] Cavalleri D, Murphy M, Seewald W, Drake J, Nanchen S. Two randomized, controlled studies to assess the efficacy and safety of lotilaner (Credelio™) in preventing *Dermacentor reticulatus* transmission of *Babesia canis* to dogs. Parasit Vectors. 2017;10:520. 29089056 10.1186/s13071-017-2473-1 PMC5664908

[30] Beugnet F, Halos L, Larsen D, Labuschagné M, Erasmus H, Fourie J. The ability of an oral formulation of afoxolaner to block the transmission of *Babesia canis* by *Dermacentor reticulatus* ticks to dogs. Parasit Vectors. 2014;7:283. 24957215 10.1186/1756-3305-7-283 PMC4078974

[31] Tielemans E, Rautenbach C, Viljoen A, Beugnet F. Efficacy of an oral combination of afoxolaner and milbemycin oxime for the prevention of transmission of *Babesia canis* by *Dermacentor reticulatus* ticks to dogs. Parasit Vectors. 2025;18:142. 40234985 10.1186/s13071-025-06787-y PMC12001589

[32] Taenzler J, Liebenberg J, Roepke RK, Heckeroth AR. Prevention of trans mission of *Babesia canis* by *Dermacentor reticulatus* ticks to dogs after topical administration of fluralaner spot-on solution. Parasit Vectors. 2016;9:234. 27241120 10.1186/s13071-016-1481-x PMC4886439

[33] Chiummo R, Zschiesche E, Capári B, Farkas R, Chiquet M, Rapti D, et al. Field efficacy of fluralaner (Bravecto® chewable tablets) for preventing *Babesia canis* infection transmitted by *Dermacentor reticulatus* ticks to dogs. Parasit Vectors. 2023;16:252. 37501160 10.1186/s13071-023-05820-2 PMC10373369

[34] Geurden T, Six R, Becskei C, Maeder S, Lloyd A, Mahabir S, et al. Evaluation of the efficacy of sarolaner (Simparica^®^) in the prevention of babesiosis in dogs. Parasit Vectors. 2017;10:415. 28877743 10.1186/s13071-017-2358-3 PMC5588728

[35] Borowski S, Viljoen A, D’Hanis L, Mahabir S, Geurden T. Evaluation of the efficacy of Simparica Trio^®^ in the prevention of the transmission of *Babesia canis* by infected *Dermacentor reticulatus* to dogs. Parasit Vectors. 2024;17:51. 38308372 10.1186/s13071-023-06115-2 PMC10836058

[36] Cavalleri D, Murphy M, Seewald W, Drake J, Nanchen S. Assessment of the speed of flea kill of lotilaner (Credelio™) throughout the month following oral administration to dogs. Parasit Vectors. 2017;10:529. 29089019 10.1186/s13071-017-2466-0 PMC5664906

[37] Cavalleri D, Murphy M, Seewald W, Drake J, Nanchen S. Assessment of the onset of lotilaner (Credelio™) speed of kill of fleas on dogs. Parasit Vectors. 2017;10:521. 29089066 10.1186/s13071-017-2474-0 PMC5664436

[38] Murphy M, Cavalleri D, Seewald W, Drake J, Nanchen S. Laboratory evaluation of the speed of kill of lotilaner (Credelio™) against *Ixodes ricinus* ticks on dogs. Parasit Vectors. 2017;10:541. 29089039 10.1186/s13071-017-2467-z PMC5664588

[39] Toutain CE, Seewald W, Jung M. The intravenous and oral pharmacokinetics of lotilaner in dogs. Parasit Vectors. 2017;10:522. 29089051 10.1186/s13071-017-2475-z PMC5664907

[40] Cavalleri D, Murphy M, Seewald W, Drake J, Nanchen S. Laboratory evaluation of the efficacy and speed of kill of lotilaner (Credelio™) against *Ixodes ricinus* ticks on cats. Parasit Vectors. 2018;11:413. 30001731 10.1186/s13071-018-2968-4 PMC6044019

[41] Cavalleri D, Murphy M, Seewald W, Nanchen S. A randomized, controlled field study to assess the efficacy and safety of lotilaner (Credelio™) in controlling ticks in client-owned cats in Europe. Parasit Vectors. 2018;11:411. 30001746 10.1186/s13071-018-2967-5 PMC6043961

[42] Murphy M, Garcia R, Karadzovska D, Cavalleri D, Snyder D, Seewald W, et al. Laboratory evaluations of the immediate and sustained efficacy of lotilaner (Credelio™) against four common species of ticks affecting dogs in North America. Parasit Vectors. 2017;10:523. 29089057 10.1186/s13071-017-2476-y PMC5664823

[43] Cavalleri D, Murphy M, Gorbea RL, Seewald W, Drake J, Nanchen S. Laboratory evaluations of the immediate and sustained effectiveness of lotilaner (Credelio™) against three common species of ticks affecting dogs in Europe. Parasit Vectors. 2017;10:527. 29089050 10.1186/s13071-017-2477-x PMC5664927

[44] Cavalleri D, Murphy M, Seewald W, Drake J, Nanchen S. A randomized, controlled study to assess the efficacy and safety of lotilaner (Credelio™) in controlling ticks in client-owned dogs in Europe. Parasit Vectors. 2017;10:531. 29089058 10.1186/s13071-017-2478-9 PMC5664821

[45] Lasmar PVF, Murphy M, Nanchen S, Drake J, Coumendouros K, Borges DA, et al. Laboratory evaluation of the efficacy of lotilaner (Credelio™) against *Amblyomma cajennense* (sensu lato) infestations of dogs. Parasit Vectors. 2018;11:537. 30285898 10.1186/s13071-018-3116-x PMC6171179

[46] Otaki H, Sonobe J, Murphy M, Cavalleri D, Seewald W, Drake J, et al. Laboratory evaluation of the efficacy of lotilaner (Credelio™) against *Haemaphysalis longicornis* infestations of dogs. Parasit Vectors. 2018;11:448. 30071885 10.1186/s13071-018-3032-0 PMC6090816

[47] Baker K, Ellenberger C, Murphy M, Cavalleri D, Seewald W, Drake J, et al. Laboratory evaluations of the 3-month efficacy of oral lotilaner (Credelio™) against experimental infestations of dogs with the Australian paralysis tick. *Ixodes holocyclus* Parasit Vectors. 2018;11:487. 30157914 10.1186/s13071-018-3061-8 PMC6116354

[48] Cavalleri D, Murphy M, Seewald W, Nanchen S. Laboratory evaluation of the efficacy and speed of kill of lotilaner (Credelio™) against *Ctenocephalides felis* on cats. Parasit Vectors. 2018;11:408. 30001727 10.1186/s13071-018-2972-8 PMC6043949

[49] Cavalleri D, Murphy M, Seewald W, Nanchen S. A randomized, controlled field study to assess the efficacy and safety of lotilaner (Credelio™) in controlling fleas in client-owned cats in Europe. Parasit Vectors. 2018;11:410. 30001744 10.1186/s13071-018-2971-9 PMC6044040

[50] Chappell K, Paarlberg T, Seewald W, Karadzovska D, Nanchen S. A randomized, controlled field study to assess the efficacy and safety of lotilaner flavored chewable tablets (Credelio™ CAT) in eliminating fleas in client-owned cats in the USA. Parasit Vectors. 2021;14:127. 33648556 10.1186/s13071-021-04617-5 PMC7923547

[51] Paarlberg T, Karadzovska D, Helbig R. Efficacy of lotilaner (Credelio™) against experimentally induced infestations of the adult cat flea, *Ctenocephalides felis*, and flea eggs following oral administration to cats. Parasit Vectors. 2021;14:139. 33673848 10.1186/s13071-021-04660-2 PMC7934462

[52] Young L, Karadzovska D, Wiseman S, Helbig R. Efficacy of lotilaner (Credelio™) against the adult cat flea, *Ctenocephalides felis* and flea eggs following oral administration to dogs. Parasit Vectors. 2020;13:25. 31937370 10.1186/s13071-019-3873-1 PMC6961367

[53] Cavalleri D, Murphy M, Seewald W, Drake J, Nanchen S. A randomised, blinded, controlled field study to assess the efficacy and safety of lotilaner tablets (Credelio™) in controlling fleas in client-owned dogs in European countries. Parasit Vectors. 2017;10:526. 29089065 10.1186/s13071-017-2479-8 PMC5664837

[54] Karadzovska D, Chappell K, Coble S, Murphy M, Cavalleri D, Wiseman S, et al. A randomized, controlled field study to assess the efficacy and safety of lotilaner flavored chewable tablets (Credelio™) in eliminating fleas in client-owned dogs in the USA. Parasit Vectors. 2017;10:528. 29089063 10.1186/s13071-017-2469-x PMC5664423

[55] Snyder DE, Wiseman S, Liebenberg JE. Efficacy of lotilaner (Credelio™), a novel oral isoxazoline against naturally occurring mange mite infestations in dogs caused by *Demodex* spp. Parasit Vectors. 2017;10:532. 29089049 10.1186/s13071-017-2472-2 PMC5664441

[56] EMEA. VICH GL9 (GCP): Guideline on Good Clinical Practices. CVMP/VICH/595/98-FINAL. 2000. https://www.ema.europa.eu/en/documents/scientific-guideline/vich-gl9-good-clinical-practices-step-7_en.pdf. Accessed 03 Feb 2026.

[57] EMA. Guideline for the testing and evaluation of the efficacy of antiparasitic substances for the treatment and prevention of tick and flea infestation in dogs and cats – Revision 4. EMEA/CVMP/EWP/005/2000-Rev.4. 2021. https://www.ema.europa.eu/en/documents/scientific-guideline/guideline-testing-and-evaluation-efficacy-antiparasitic-substances-treatment-and-prevention-tick-and-flea-infestation-dogs-and-cats-revision-4_en.pdf. Accessed 03 Feb 2026.

[58] EMA. Guideline on data requirements for veterinary medicinal products intended to reduce the risk of transmission of vector-borne pathogens in dogs and cats. EMA/CVMP/EWP/278031/2015. 2022. https://www.ema.europa.eu/en/documents/scientific-guideline/guideline-data-requirements-veterinary-medicinal-products-intended-reduce-risk-transmission-vector-borne-pathogens-dogs-and-cats_en.pdf. Accessed 03 Feb 2026.

[59] Marchiondo AA, Holdsworth PA, Green P, Blagburn BL, Jacobs DE. World association for the advancement of veterinary parasitology (W.A.A.V.P.) guidelines for evaluating the efficacy of parasiticides for the treatment, prevention and control of flea and tick infestation on dogs and cats. Vet Parasitol. 2007;145:332–44. 17140735 10.1016/j.vetpar.2006.10.028

[60] Otranto D, Dantas-Torres F, Fourie JJ, Lorusso V, Varloud M, Gradoni L, et al. World association for the advancement of veterinary parasitology (W.A.A.V.P.) guidelines for studies evaluating the efficacy of parasiticides in reducing the risk of vector-borne pathogen transmission in dogs and cats. Vet Parasitol. 2021;290:109369. 33548595 10.1016/j.vetpar.2021.109369

[61] Troskie M, de Villiers L, Leisewitz A, Oosthuizen MC, Quan M. Development and validation of a multiplex, real-time PCR assay for *Babesia rossi* and *Babesia vogeli*. Ticks Tick Borne Dis. 2019;10:421–32. 30591405 10.1016/j.ttbdis.2018.12.004

[62] SAS Institute Inc. (2025). SAS software (Version 9.4).

[63] Kidd L, Breitschwerdt EB. Transmission times and prevention of tick-borne diseases in dogs. Comp Cont Educ Pract Vet. 2003;10:742–51.

[64] Otranto D. Arthropod-borne pathogens of dogs and cats: from pathways and times of transmission to disease control. Vet Parasitol. 2018;251:68–77. 29426479 10.1016/j.vetpar.2017.12.021

[65] Wenger MJ, Kollasch TM, Burke MC, Jones L, Locklear C, Hedberg M, et al. Early onset of pre-lethal effects of lotilaner (Credelio®) on* Amblyomma americanum* ticks on experimentally infested dogs. Parasit Vectors. 2021;14:322. 34120646 10.1186/s13071-021-04817-z PMC8201672

